# Diagnostic features of *Acanthamoeba* keratitis via *in vivo* confocal microscopy

**DOI:** 10.1038/s41598-025-94567-0

**Published:** 2025-03-29

**Authors:** Joanna Przybek-Skrzypecka, Malcolm Armstrong, Jennifer Kim, Andrew Walkden, Leon Au, Arun Brahma, Fiona Carley, Jaya Devi Chidambaram

**Affiliations:** 1 Department of Ophthalmology, Medical University of Warsaw, Warsaw, Poland; 2 Cornea Department, Manchester Royal Eye Hospital, Manchester, UK; 3 Microbiology Department, Manchester Royal Infirmary, Manchester, UK; 4 School of Pharmacy & Optometry, Faculty of Biology, Medicine and Health, University of Manchester, Manchester, UK

**Keywords:** In vivo confocal microscopy, Keratitis, *Acanthamoeba*, Cyst, Corneal ulcer, Diagnosis, Biomarkers, Corneal diseases

## Abstract

*In vivo* confocal microscopy (IVCM) offers a non-invasive, rapid method for diagnosing *Acanthamoeba* keratitis (AK) by detecting cysts or trophozoites in the initial clinic visit images. In this retrospective observational study, we reviewed HRT3 IVCM images from patients presenting to Manchester Royal Eye Hospital with clinically- suspected AK for IVCM morphological features (IVCM-MF) of both *Acanthamoeba* and corneal cells. Twenty-seven patients were included in the study: median age 29 years (range 16–71 years), female gender (59%; n = 16/27) and contact lens wear as the main risk factor. Median symptom duration before the initial ophthalmologist visit was 9 days (range 2 to 42 days). IVCM had a higher detection rate for AK in 85% of patients (n = 23/27), with culture positivity in only 74% (n = 20/27; 17 of whom were also IVCM-positive). *Acanthamoeba* IVCM-MF included: bright spots (87%, n = 20/23), double-walled cysts (56%, n = 13/23), signet-ring (22%, n = 5/23) and trophozoites (30%, n = 7/23). Bright spots and double-walled cysts coalesced in lines/clusters in 1 patient. Corneal epithelial cells had a “koilocyte” appearance in 64% (n = 14/22). Microtubules connecting adjacent keratocytes were visible in 52% (n = 12/23), particularly associated with *A. polyphaga* ulcers (p = 0.02). These IVCM features observed in corneal epithelial cells and keratocytes may represent potential imaging biomarkers for AK diagnosis and warrant further investigation to validate their diagnostic utility. By demonstrating IVCM’s superior diagnostic performance, providing rapid and accurate diagnostics, this study advocates for its inclusion in standard diagnostic workflows for AK, paving the way for future advancements in clinical practice.

## Introduction

*Acanthamoeba* keratitis (AK) is a vision-threatening condition often missed in early diagnosis due to its atypical presentation and limitations in conventional diagnostic methods. AK is a serious sight-threatening ocular disease that causes poor vision outcomes in ~ 50% of patients^1^. Its incidence is increasing worldwide mostly due to increasing contact lens wear^2^. Early diagnosis in the course of AK is critical, as this brings better visual outcomes by enabling the clinician to be able to start correct antimicrobial therapy and thereby prevent the ensuing vision-threatening tissue damage^3^. The current standard of care involves a complex diagnostic journey for the patient, with each step in this journey having a moderate to low diagnostic accuracy for AK: slit lamp examination to look for typical AK features (many of which are rarely observed at the onset of disease) such as perineural infiltrates, dendritiform ulcer appearance, ring infiltrates; corneal scraping for microbiological stains/cultures/PCR (limited due to only being able to scrape from the superficial cornea and time delays for culture results or access to PCR testing)^4^. Scanning electron microscopy (SEM) has proven useful in detecting *Acanthamoeba* sp. in scrapings from the conjunctiva of the everted upper lid of patients with culture-negative microbial keratitis^5^. This technique may serve as a diagnostic method in clinics with access to SEM and to the expertise for interpretation of the organism morphology in the SEM images. Further time delay and tissue damage may occur if AK is not suspected initially, and the patient may present many days later after treatment failure with initial antibiotics/antifungals.

*In vivo* confocal microscopy (IVCM) is a rapid, non-invasive imaging modality that can aid the clinician to accurately diagnose AK, and other corneal diseases, at the initial clinic visit, thereby potentially reducing the time to correct diagnosis, start of appropriate antimicrobial therapy and potentially better vision outcomes^6,7^. Many publications have identified the main diagnostic imaging biomarker within IVCM images to be the presence of *Acanthamoeba* cysts or trophozoite morphologies^8,9^. *Acanthamoeba* cysts have well-defined IVCM morphological features (IVCM-MF): double-walled cysts, the “bright spot” sign, and the “signet ring” sign^10,11^. Detection of *Acanthamoeba* cysts in IVCM images at the first clinic visit can have a high sensitivity and specificity for diagnosis of AK^6,9^. These publications have also highlighted the imaging limitations, namely, the difficulties sometimes of identifying cysts due to overlap in appearance to other structures such as white cells, and also the variability in appearance of cysts and trophozoites^10,12^. Very few papers have looked at the other corneal cellular and tissue IVCM-MF that co-occur with cysts/trophozoites, that might additionally help with confirming the diagnosis of AK^12^. The use of clusters of spatially co-occurring imaging biomarkers at the site of disease has been increasingly shown to enhance diagnostic accuracy across various therapeutic areas, such as oncology, where the complexity of the tumour microenvironment allows for more precise cellular and morphological diagnoses when imaging features are assessed collectively^13^.

This study aims to evaluate the diagnostic value of novel imaging biomarkers detected via IVCM, a tool not widely applied in routine practice. We hypothesize that IVCM imaging could reveal additional features within the cornea beyond presence or absence of *Acanthamoeba* cysts/trophozoites, and that these could be used to aid in diagnosis of AK. We have assessed IVCM images of patients presenting to the Manchester Royal Eye Hospital (MREH) cornea clinic with clinically-suspected AK, to look for *Acanthamoeba* and corneal IVCM-MF. We also assessed associations with culture results (including *Acanthamoeba* subspeciation), as well as clinical signs observed in slit lamp biomicroscopy.

## Materials and methods

This retrospective, non-interventional study was conducted in accordance with the Declaration of Helsinki. Ethical approval was obtained from the UK National Integrated Research Application System (IRAS). Due to the retrospective nature of this study and the use of anonymous, unidentifiable patient data, the requirement for informed consent to participate was waived by the UK Health Research Authority and UK National Health Service Research Ethics Committees. All patients presenting to the Cornea Department at MREH in the UK, between 2012 and 2018 with clinically-suspected *Acanthamoeba* keratitis (AK) were included in this study. Socio-demographic and clinical history data obtained from casenote review included: age, gender, contact-lens usage, time from the first symptoms to clinical diagnosis of AK; any steroid treatment started prior to clinical diagnosis of AK, treatment modes (antibiotics, antiviral, antifungal, anti-amoebal drugs), visual acuity at MREH visit and slit-lamp examination findings.

### Diagnosis of AK

**Fig. 1:**
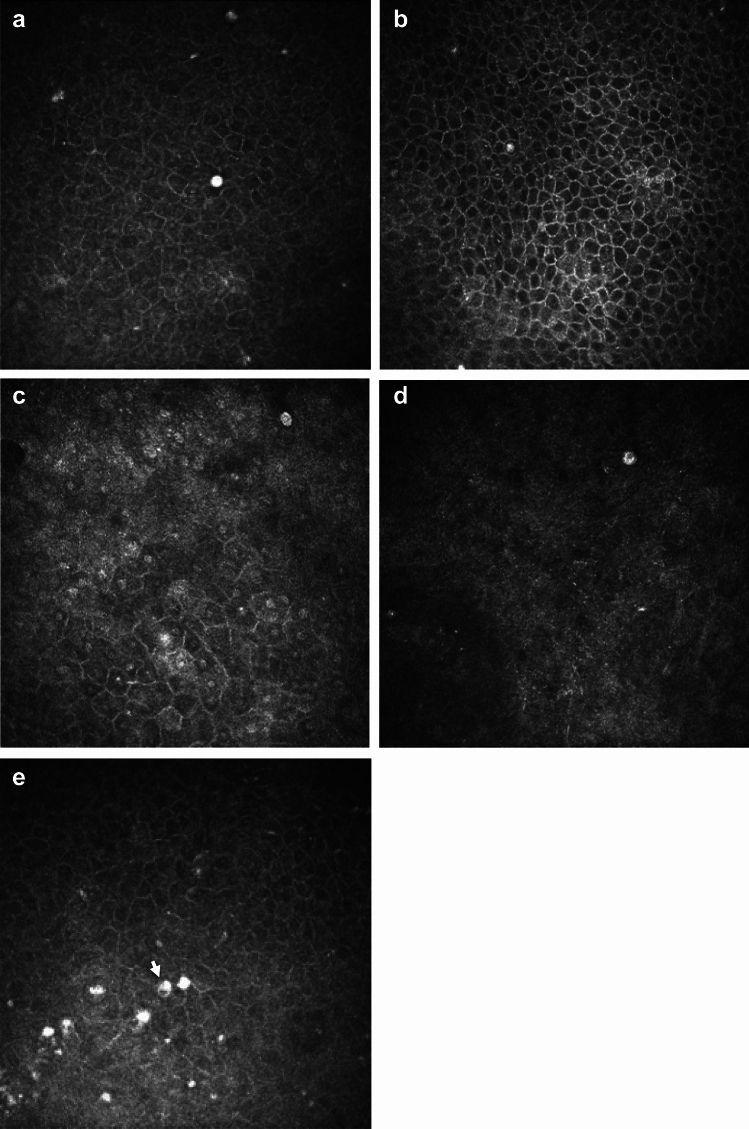
*Acanthamoeba* cysts morphology on *In Vivo* Confocal Microscopy imaging. (**a**) Bright spot cyst appearance in the center of the image (approximately 10 microns in diameter, characterized by highly reflective, uniformly white appearance, and very circular outline of cyst). (**b**) Small bright spot cyst (< 10 microns in diameter) which appears to be present wholly within an epithelial cell (i.e. dark cytoplasm observed around bright spot, and bright cell border then seen to encompass cytoplasm and bright spot). This bright spot cyst has less uniform hyper-reflectivity compared to spot in (Fig. 1a). (**c**) Double walled cyst with bright center (top right corner of image). Cyst appears to have white hyper-reflective centre, with dark surround and finally, white hyper-reflective outer wall. (**d**) Double walled cyst with dark center (“empty cyst”). Cyst appears to have a corrugated outer wall, and dark center which is slightly off-set. (**e**) “Signet ring” cyst appearance shown by arrow, and seen to the left side of bright spot cyst.

**Fig. 2:**
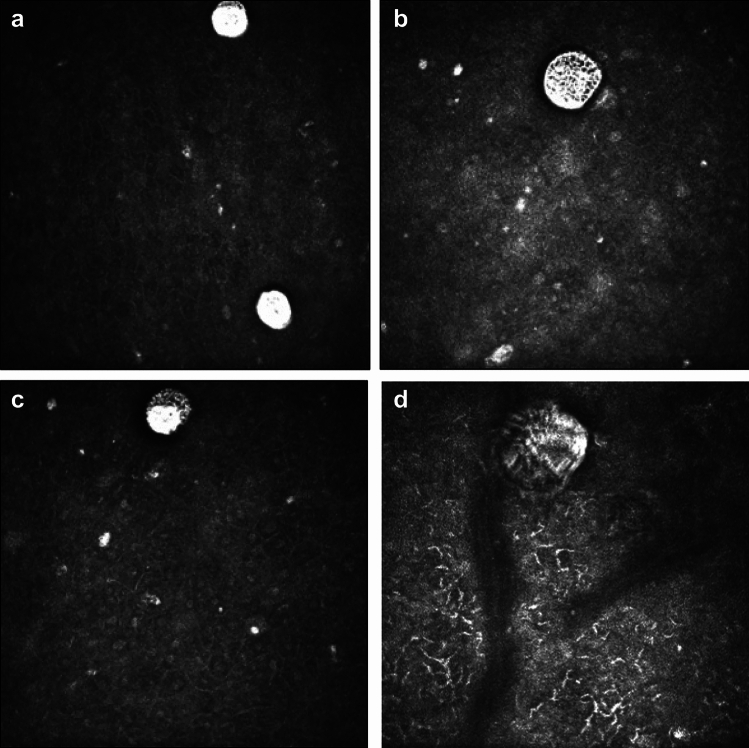
Trophozoite IVCM morphological features (IVCM-MF) seen in *In Vivo* Confocal Microscopy images of *Acanthamoeba* keratitis. (**a**) Trophozoites with overall large bright spot appearance and dark halo surround observed. Central nuclear outline faintly observed within trophozoite at top of image. Faint dappled appearance observed within trophozoite at bottom of image. (**b**) Trophozoite with dappled appearance and dark halo surround. (**c**) Trophozoite with dappled surface, large bright hyper-reflective nucleus and dark surround. (**d**) Trophozoite in Bowman’s layer. Bowman’s membrane linear broad collagen lamellae seen to be traversing image and one collagen bundle noted adjacent to trophozoite). Trophozoite with irregular surface, dark halo and bright irregular outer cell wall surround. Small bright curvilinear dendriform cells appear in anterior stroma.

Corneal scrapes were cultured on *Escherichia coli*-enriched agar in Microbiology Department of Manchester Royal Infirmary. *Acanthamoeba* species were identified based upon cyst morphology at the *Acanthamoeba* keratitis reference unit in the Diagnostic Parasitology Laboratory, London School of Hygiene and Tropical Medicine. Diagnosis of *Acanthamoeba* keratitis (AK) was made by presence of one of the following: 1) microbiological culture positivity for *Acanthamoeba* cyst or trophozoite; 2) observation of *Acanthamoeba* cyst or trophozoite appearances in IVCM imaging (reference images from prior publications were used to aid in identification of various cyst^12,14^ and trophozoite^15^ morphologies; see Figs. 1 and 2 for IVCM images representative of cyst and trophozoite morphologies); or 3) clinical suspicion of *Acanthamoeba* keratitis based on slit lamp examination findings, followed by commencement of anti-amoebal treatment and improvement of clinical features after use of anti-amoebal treatment.

### IVCM image acquisition and image grading

All patients underwent *in vivo* confocal microscopy imaging (IVCM) of corneal ulcer in the Cornea Service MREH, using the HRT3 IVCM with Rostock corneal module (Heidelberg Engineering, Heidelberg, Germany). Patients had both eyes anesthesized with preservative-free 0.5% proxymetacaine eye drops (Minims, Bausch & Lomb, UK) prior to IVCM imaging. Volume scan mode was used for automated acquisition of 40 consecutive series of adjacent images covering 80 microns total thickness of the corneal depth (each image measuring 400 × 400 μm in the X-Y axis), with the operator manually refocusing to capture regions of interest in the ulcer between epithelium to endothelium. In some patients, single section scans (400 × 400 microns single scans in the X-Y axis) were also obtained to document presence of specific features such as *Acanthamoeba* cysts or trophozoite appearances. All IVCM imaging was performed by personnel with experience in IVCM image acquisition (either corneal nurse practitioners or an ophthalmologist). No information was available on the order of testing, and whether IVCM had been performed before or after corneal scraping for microbiological testing. All IVCM images had patient-identifying data and microbiological result data removed, prior to being graded. Two experienced ophthalmologists (JDC and JP-S) reviewed IVCM images, focusing on cyst morphologies, trophozoites, and corneal changes. Grading results were entered into an Excel spreadsheet (Microsoft).

Presence or absence of specific IVCM-MF was evaluated in the entire IVCM image-set. For *Acanthamoeba* cysts, the following were assessed: double-walled cyst, “bright spot” sign, “signet ring” sign, cysts appearing together in lines or clusters, and trophozoite appearances. For the cornea, the following IVCM-MF were evaluated: epithelial and endothelial cells (size/shape/nuclear versus cytoplasm reflectivity; cell border appearances); corneal nerves (beading/tortuosity in the basal nerve fibre layer plexus); keratocytes (normal or activated appearances) and dendritiform cells/other inflammatory cells (tissue location, size, density) as observed in either anterior stroma (defined as images covering a maximum depth of 250 microns from the superficial-most corneal epithelial image), or posterior cornea (images covering > 250 microns depth in the stroma through to endothelial cells).

### Statistical analysis

Analyses was performed in Stata 13.1 (StataCorp, College Station, Texas, USA). The ordinal grading method was applied (presence/absence of IVCM-MF). Data extracted in the study were analysed using logistic regression, or for group comparisons, chi-squared testing or fisher’s exact test was used. P-values less than 0.05 were considered statistically significant.

## Results

During the study period of December 2012 to November 2018, 27 patients were identified who presented to the MREH cornea clinic with clinically-suspected AK and had IVCM imaging performed. Baseline socio-demographic features of these patients are summarized in (Table 1). Of note, the main risk factor identified was contact lens (CL) wear, present in all 21 patients for whom this data was available.

**Table 1:** Baseline socio-demographic and clinical characteristics of patients.

Age, median in years (range)	29 (16–71)
Gender, female, n (%)	16/27 (59.3%)
Time from symptom onset to seeing ophthalmologist, median time in days (range)	9 (2–42)
Contact lens-wearer as risk factor*	21/21 (100%)
Best-corrected visual acuity at first cornea clinic visit, median logMAR (range)	0.25 (0.0-Hand Movements)

*missing data for 6 patients.

AK diagnosis results based on IVCM, culture and clinical treatment response to anti-amoebal therapy are shown in (Table 2). The modality with greatest detection rate for diagnosis of AK was IVCM with 85% of patients (n = 23/27) having *Acanthamoeba* cyst and/or trophozoite morphologies observed in IVCM imaging. Microbiological culture had a detection rate of 74% (n = 20/27). *Acanthamoeba* subspecies were identified in 16 out of the 20 cultures as follows: *A. polyphaga* in 10, *A. astronyxis* in 3, *A. hatchetti* in 2, *A. castellani* in 1. Of note, in the 22% of patients (n = 6/27) who were culture negative, the diagnosis of AK was determined using IVCM positivity for *Acanthamoeba* cysts or trophozoite appearances.

**Table 2:** Diagnostic modalities used for *Acanthamoeba* keratitis and test results.

Diagnostic test	Culture positive	Culture negative	Total
IVCM positive	17	6	23
IVCM negative	3	1*	4
Total	20	7	27

*The culture-negative and IVCM-negative patient was diagnosed based on clinical signs at diagnosis and also had clinical signs of treatment response to anti-amoebal therapy.

One patient was both IVCM and culture negative for *Acanthamoeba* had the AK diagnosis made based on presence of AK signs in slit lamp biomicroscopy along with clinical improvement after anti-amoebal therapy.

### *Acanthamoeba* cyst and trophozoite IVCM-MF

Of the 23 patients who were positive for *Acanthamoeba* cyst/trophozoite IVCM-MF*,* a variety of cyst and trophozoite appearances were detected (data shown in Supplemental Table 1), as summarized in (Fig. 1). The “Bright spot” morphology (see Fig. 1a) was the most frequent to be observed in 87% of patients (n = 20/23). Bright spots most often appeared as hyper reflective, circular objects, approximately 10 microns in diameter, however, occasionally the bright spots appeared with smaller diameter and polygonal shape, rather than circular. The bright spots were organized in linear chains as well as clusters in 1 patient only, and this patient was noted to be using topical steroids before their initial MREH cornea clinic visit. Bright spot cysts were occasionally observed to lie entirely inside an intact epithelial cell border as observed in 7 patients (Fig. 1b).

Double-walled cyst appearances were the next most commonly observed cyst morphology, seen in 56% of patients (n = 13/23). Ten patients had both bright spot and double-walled cysts co-occurring within their IVCM image-sets. Double walled cysts were observed to have either a bright center (n = 3) or a dark center (n = 10), hereafter termed “empty cysts” (shown in Fig. 1c,d respectively). Patients with “empty cysts” had a slightly longer symptom duration prior to attending the MREH cornea clinic than those with double walled cysts with bright centers (median 14 days (IQR 7–15 days) compared with 9 days (IQR 7–15 days)), although this difference was not statistically significant (p = 0.56). The two patients who were using topical steroids at presentation, both had “empty cysts” that co-occurred with bright spots, and one of the patients additionally had bright-centered double wall cysts observed as well. Five patients were noted to have a “signet ring” appearance of cysts (Fig. 1e).

Trophozoite IVCM-MF were detected less often and were only seen in 7 of the 23 patients with IVCM positivity for *Acanthamoeba* appearances. The morphology of trophozoites was greatly variable as shown in Fig. 2a–d. Sizes ranged from ~ 50 to 100 μm diameter. The IVCM appearances included hyper-reflective round/ovoid structures (Fig. 2a) with surface dappled appearance (Fig. 2b,c), surrounded by a dark halo, and sometimes with an additional outer wall beyond the dark halo (Fig. 2d). Trophozoites co-occurred with bright spots in all 7 patients and with double walled cysts in 6 patients (of whom 5 had “empty cyst” double-walled appearances).

### Corneal epithelial cell layer IVCM-MF

**Fig. 3:**
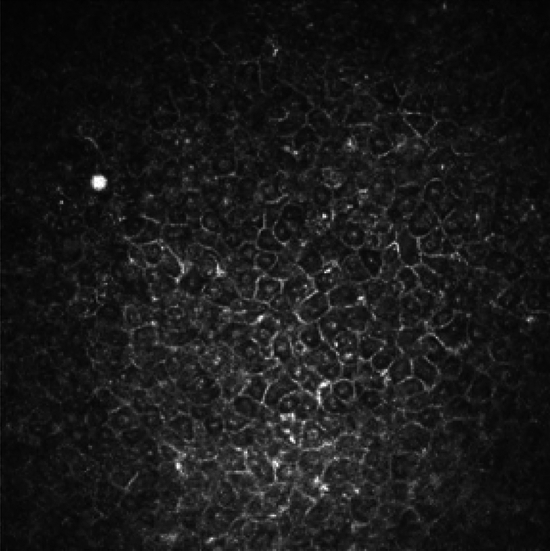
Bright spot cyst (uniformly hyper-reflective) associated with corneal epithelial “Koilocyte” appearance: dark perinuclear cytoplasm “halo”, hyper-reflective nuclei and hyper-reflective cell borders in epithelial cells.

Corneal epithelial cells showed a pattern of distinct morphologies that were observed in the 22 patients in whom the epithelium was visualized in IVCM images. The most frequent epithelial IVCM-MF seen in 64% of patients (n = 14/22) was a combination of features in the cell: perinuclear hypo-reflective dark “halo”, gray cytoplasm and often with a bright hyper-reflective cell border (hereafter referred to as “koilocyte” appearance) as shown in (Fig. 3). Presence of “bright spot” cysts, was associated with this koilocyte appearance (n = 12/22, p = 0.04). We also observed in 6 patients, patches of cells within the en face image plane to have heterogenous cell sizes, with some cells displaying an expanded diameter compared with other adjacent smaller cells. Finally, large ovoid / circular regions with uniform low reflectivity within them (herein termed as “bullae”), and surrounded by intact epithelial cells with bright borders were noted in a few patients (27%, n = 6/22).

**Fig. 4:**
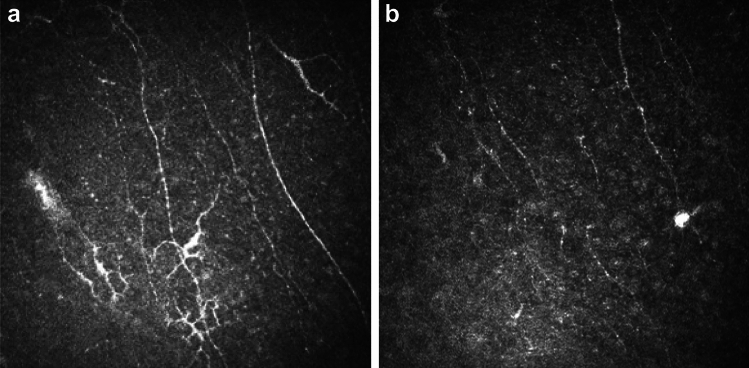
Corneal nerve IVCM morphologies observed in *Acanthamoeba* keratitis. (**a**) Beading of Corneal Nerves in basal nerve plexus (mostly associated with *Acanthamoeba polyphaga* infection). Nerves appear to have a “string of pearls” appearances in some regions. Prominent dendritiform cells observed along corneal nerves, with long processes extending outwards. (**b**) Double walled cyst with bright center seen along beaded corneal nerve in basal plexus.

Corneal nerve fibers in the basal plexus (between the basal layer of corneal epithelial cells and Bowman’s membrane) were noted to have a beaded appearance in 6 patients (see Fig. 4), but were often not visible within the basal plexus images (i.e. in 9 patients). Beaded nerves were associated with culture-positivity for *A. polyphaga* (p = 0.03).

Dendritiform cells with large cell bodies and prominent elongated cellular processes, were visible in the basal corneal epithelial layer and/or adjacent to the basal nerve plexus in 10 patients (Fig. 4a). Eight patients had smaller dendritiform cells in the anterior stroma immediately beneath Bowman’s membrane, many of which appeared to have short cellular processes interconnected with neighbouring dendritiform cells.

### Corneal stromal and endothelial IVCM-MF

**Fig. 5:**
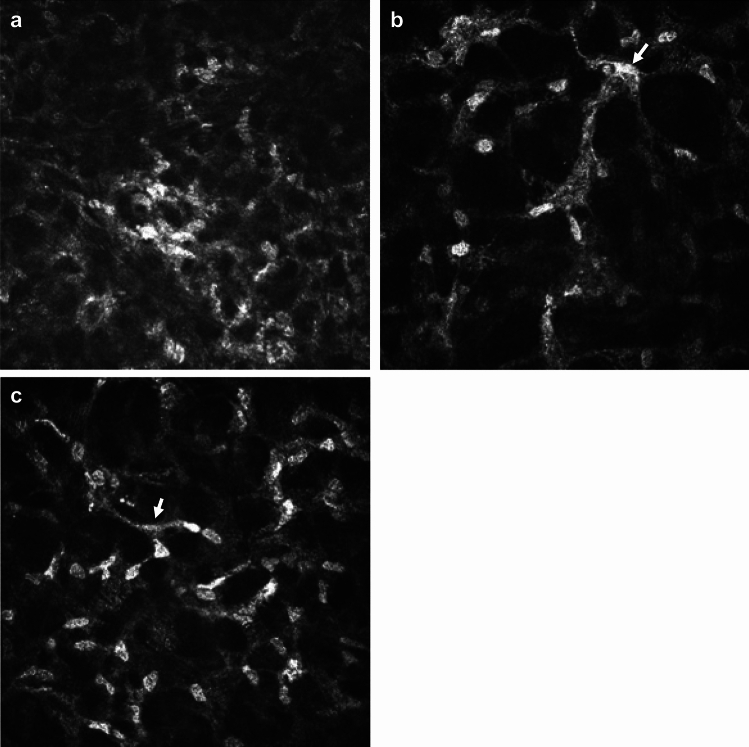
Corneal stroma. (**a**) Keratocytes with “activated” appearance: hyperreflective ovoid nuclei and hyper-reflective interconnected cell processes (“honeycomb appearance”). (**b**) Bright hyper-reflective “connections” within cellular processes of adjacent keratocytes as shown by arrow. (**c**) Linear “microtubule” connections seen between adjacent keratocyte processes (arrow).

An activated keratocyte appearance was observed in anterior and/or posterior stroma in 78% of patients (n = 18/23). Curvilinear connections between adjacent keratocyte nuclei were observed in 56% of patients (n = 13/23) as shown in (Fig. 5). Adjacent keratocytes throughout the stroma were often visibly connected by hyper-reflective linear regions within the keratocyte processes (n = 12/23, named “microtubules” hereafter), and this IVCM-MF was associated with *A. polyphaga* culture positivity (p = 0.02). Several other stromal IVCM-MF were seen albeit in smaller numbers of patients: 7 patients with linear, hyper-reflective, bright non-branching structures in the stromal extracellular matrix, often grouped in multiples arranged in parallel to each other (named “spindles”); 5 patients had the appearance of prominent wide diameter nerve fiber bundles seen in the corneal stroma; 4 patients were seen to have “ghost” keratocyte appearances in the anterior and posterior stroma (i.e. keratocytes with broad cellular processes but no visible nuclei); 1 patient with a linear arrangements of bright spots along keratocyte processes forming a honeycomb appearance.

Very few endothelial abnormalities were detected on IVCM imaging. One patient was found to have a hyper-reflective endothelial cell nuclei. Two patients were found to have dendritiform cells arranged on the posterior aspect of the endothelial cell mono-layer. The data for IVCM-MF graded in the corneal epithelium, stroma and endothelium are shown in Supplemental Table 2.

### IVCM-MF and slit lamp examination findings

The classic slit lamp sign of a “dendritic” ulcer was observed in only 2 patients overall, both of whom were IVCM positive for AK (1 culture positive, 1 culture negative). The IVCM-MF seen in both patients were bright spots co-occurring with double-walled cysts, and the “koilocyte” appearance of epithelial cells. For another classical slit lamp sign of AK, the ring infiltrate, 3 patients presented with this sign, all of whom were IVCM positive for AK (two were additionally culture positive for AK). IVCM-MF in these patients included bright spots, double-walled “empty cysts”, and trophozoites observed to be co-occurring in the images. The final AK slit lamp sign that was observed were peri-neural infiltrates that were seen in 8 patients, all of whom were culture positive for AK, but only 7 of whom were IVCM positive. Their IVCM-MF included mainly bright spots in 6 patients, double walled cysts in 3 (bright or dark centered), and trophozoites in 2. Corneal nerve appearances in the IVCM images of these patients showed prominent stromal nerves in 3 patients and beaded nerves in the basal plexus in 3 patients. Double-walled cysts were detected immediately adjacent to the prominent stromal nerve in 1 patient’s IVCM images.

## Discussion

*Acanthamoeba* keratitis (AK) can lead to devastating vision loss or even loss of the eye if not diagnosed and treated promptly with appropriate therapy. Previous studies have demonstrated the value of IVCM as a rapid diagnostic tool for AK at the initial clinic visit, especially when performed by operators skilled in image acquisition and clinicians proficient in interpreting AK images for accurate diagnosis. In this real-world study, we found that AK diagnosis was more frequently achieved using IVCM compared to culture positivity or the identification of pathognomonic signs through slit-lamp examination. These findings align with previous studies that show IVCM has a higher detection rate for AK cysts and trophozoites than culture alone^9,16^. In this study, we could not confirm the sequence of IVCM imaging and corneal scraping; if scraping was performed first, this may have limited the ability to detect *Acanthamoeba* cysts or trophozoites in IVCM imaging in some patients. To improve diagnostic accuracy for microbial keratitis, diagnostic pathways involving IVCM should specify the testing order, recommending IVCM imaging before corneal scraping to maximize the chance of visualizing organisms in situ on the corneal surface. Additionally, IVCM is particularly valuable for deep-seated infections where superficial corneal scraping may fail to detect the organism, resulting in a negative microbiological culture results.

In this study, we observed various IVCM-MF of *Acanthamoeba* cysts, including bright spots, double-walled cysts with bright or dark centers, and signet ring appearances. Similar morphologies *of *in vivo *Acanthamoeba* cysts have been reported by Alantary et al., who confirmed the imaging findings with ex vivo studies. Several of the imaging findings in our study, bright spots with smaller diameter and polygonal shape, rather than circular, were also observed and attributed to different imaging planes through the cyst producing these variations in imaging findings^10^. Prior publications have described the appearance of trophozoites on IVCM imaging, both *in vivo* and *ex vivo*, reporting their size to range from 17 to ~ 100 microns in diameter and morphology similar to our findings, i.e. a round nucleus and dappled surface^10,11,15,17^.

When taken in the context of recent publications, our findings are similar to those of recent large clinical studies, in which IVCM imaging at diagnosis mainly reveals bright spots and/or double walled cysts, along with presence of dendritiform cells and absence of sub-basal nerves being the main diagnostic IVCM-MFs^16,18–20^. Most prior studies have primarily focused on identifying *Acanthamoeba* cyst-like structures or trophozoites in IVCM images as the key diagnostic markers for AK^20^. Diagnosing AK becomes particularly challenging when atypical cyst features appear in IVCM imaging, such as incomplete roundness, smaller diameters (< 10 microns), or reduced hyper-reflectivity, which may resemble white blood cells and create diagnostic uncertainty^18^. Limited attention has been given to the surrounding corneal cells as potential diagnostic biomarkers. In our study, we identified specific epithelial and stromal cell changes in IVCM images from patients presenting with clinically-suspected AK that may play a role as diagnostic imaging biomarkers.

Our findings indicate the presence of koilocyte-like epithelial cells in association with bright spot cyst appearances, suggesting that clinicians should consider the co-occurrence of these IVCM-MFs to heighten diagnostic suspicion for AK, especially in atypical cases. The epithelial changes observed in our study may also shed light on the underlying pathophysiology of AK. The bright epithelial borders, dark perinuclear cytoplasm, and nuclear enlargement resemble cytopathic effects seen in HPV-infected cervical epithelial cells, where the term “koilocyte” was coined^21,22^. This epithelial cell morphology has been described in the cornea in IVCM imaging of dry eye disease, especially in Sjögren’s syndrome, where the epithelial alterations co-occur with tortuosity and beading of corneal nerves in the basal plexus^23^. Perinuclear vacuoles in epithelial cells—observed in several infections—might also represent either reactive inflammatory changes of these cells or a cytopathic effect induced by the *Acanthamoeba*^21,24,25^. Further research is necessary to assess the progression of these epithelial changes and their response to treatment.

In the corneal stroma, our study’s key findings included the observation of linear hyper-reflective and c-shaped connections between keratocytes, which have been proposed in other inflammatory corneal conditions as representing the appearance of macrophages migrating between or along keratocytes^26^. This feature was more frequently observed in *A. polyphaga*-positive ulcers, suggesting the need for more research into patient outcomes and the prognostic significance of these macrophage-like IVCM-MFs for AK caused by various *Acanthamoeba* subspecies. We also found the presence of an activated keratocyte and ghost keratocyte phenotypes, in line with previous reports from AK IVCM studies^12^.

Limitations of our study include the relatively small sample size, retrospective approach and use of a data from a single center. Future prospective studies are needed to validate these findings in larger sample sizes, diverse populations, and to explore integration of IVCM with other diagnostic methods (such as slit lamp examination, PCR, next generation sequencing, corneal biopsy, Artificial Intelligence (AI)-based models, etc.). Single-center studies are often limited by biases (e.g. small sample sizes, restricted population diversity, localized clinical practices, limitations in access to diagnostic technology, or unique environmental factors) that can reduce the generalizability of their results. Conducting multicenter studies across varied demographic and clinical settings can mitigate these biases, enhancing the broader applicability and relevance of the findings. Furthermore, these studies could investigate the diagnostic performance of the imaging biomarkers that we have identified by evaluating their sensitivity and specificity. Additionally, future research should explore whether IVCM features can predict *Acanthamoeba* subspecies, which may provide further prognostic information.

Similar to the techniques employed in oncology for identification of co-occurring imaging features to enhance diagnostic and prognostic accuracy^13^, future IVCM research could focus on identifying clusters of IVCM-MFs that differentiate between various corneal infections, including bacterial, fungal, and *Acanthamoeba* keratitis. By combining imaging biomarkers—such as koilocyte morphology, macrophage presence, and cyst/trophozoite characteristics—clinicians could develop imaging-based clinical protocols to guide diagnosis and treatment. This comprehensive imaging approach would enable clinicians to more accurately assess disease progression, evaluate treatment effectiveness, and potentially predict visual outcomes in AK patients. Future studies could also investigate use of AI to automate the detection of IVCM-MFs and to quantitate the impact of incorporation of IVCM into the standard diagnostic workflow, for example, enabling a shortened time to diagnosis as well as improvement in vision outcomes^16,27,28^. Recent AI applications in real-world IVCM datasets have shown promising results, demonstrating relatively high diagnostic accuracy in cyst identification and streamlining image analysis; these AI models have been able to efficiently filter out artifacts, prioritize relevant images containing IVCM-MFs for AK, thereby providing the ability to reduce the manual workload for ophthalmologists, and improving the detection of key diagnostic features^29^. While further validation across diverse datasets is needed, AI holds significant potential for AK diagnosis.

**Fig. 6:**
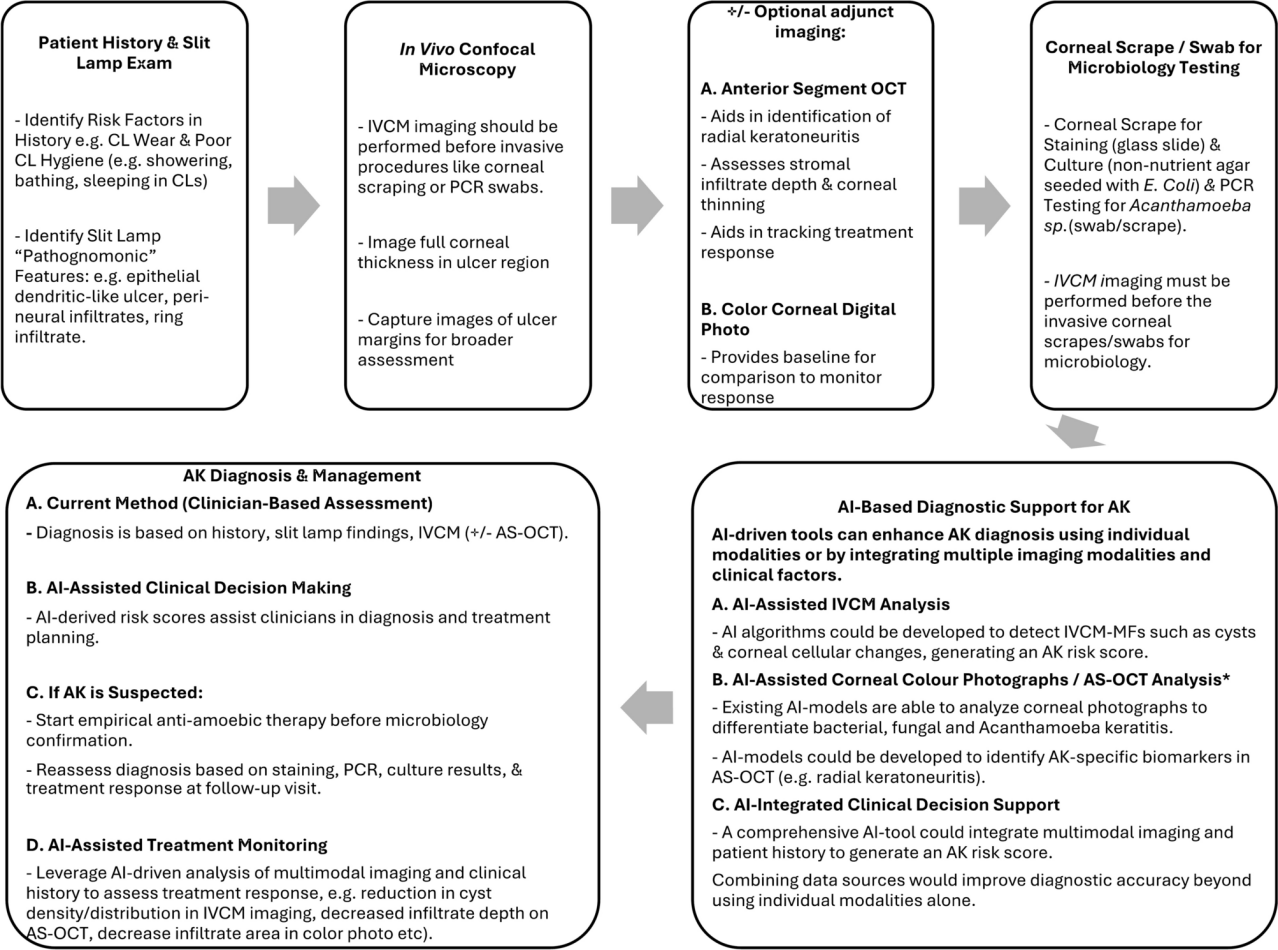
Diagnostic workflow for *Acanthamoeba* keratitis (AK), Incorporating artificial intelligence (AI)-tools.

AI-powered diagnostic workflows, such as the one illustrated in (Fig. 6), demonstrate how AI-assisted clinical decision support tools can enhance the AK diagnostic process. By integrating multimodal data—including clinical history, slit lamp findings, IVCM images, and additional imaging such as color corneal photographs and anterior-segment optical coherence tomography—AI tools can generate a comprehensive risk score, significantly improving diagnostic accuracy and efficiency. Leveraging a combination of clinical risk factors and imaging biomarkers at the initial patient visit could expedite diagnosis, facilitate earlier initiation of appropriate antimicrobial therapy for severe infections such as fungal keratitis and AK, and ultimately improve vision outcomes. We anticipate that future studies will support the establishment of IVCM as a primary diagnostic tool for microbial keratitis.

## Supplementary Information

Supplementary Information.

## Data Availability

Data obtained from grading of IVCM morphological features which form the basis of the results presented in this paper are shown in Supplemental Table 1 for *Acanthamoeba* features, and Supplemental Table 2 for corneal features. The data that support the findings of this study are available from the corresponding author (Dr. Jaya Chidambaram) on reasonable request. The findings within this paper were presented in part at the Annual Research in Visual science & Ophthalmology (ARVO) meeting in 2021^30^.
