# Supplementary material for: Diagnostic features of Acanthamoeba keratitis via in vivo confocal microscopy
Source: Sci Rep. 2025 Mar 29;15:10940. doi: 10.1038/s41598-025-94567-0 (PMC11954979; doi:10.1038/s41598-025-94567-0)
Supplement: Supplementary file 1 — Supplementary Information. [file 41598_2025_94567_MOESM1_ESM.docx]

**Supplementary data:**

**Supplementary Table 1. Morphological features of *Acanthamoeba* sp. detected within in vivo confocal microscopy (IVCM) images at diagnosis (total no. of patients=23)**

| **Morphological feature** | **No. of Patients** | **%** |
| --- | --- | --- |
| Bright spot sign | 20/23 | 87% |
| Double wall cyst | 13/23 | 56% |
| Signet ring sign | 5/23 | 22% |
| Acanthamoeba cysts observed within epithelial cells | 7/23 | 30% |
| Acanthamoeba cysts present in Bowman's layer | 1/23 | 4% |
| Trophozoite | 7/23 | 30% |
| Large bubble-like trophozoite appearance (hyperreflective surface with dark halo surround) | 6/23 | 26% |

**Supplementary Table 2: *In Vivo* Confocal Microscopy morphological features (IVCM-MF) of corneal tissue in *Acanthamoeba* keratitis patients at diagnosis**

| **Corneal location** | **IVCM-MF** | **Totals** |
| --- | --- | --- |
| **Epithelium (observed in 22 patients’ imagesets)** | “Koilocyte” appearance in epithelial cells with with peri-nuclear dark halo | 14/22 (64%) |
|  | Hyper-reflective border of epithelial cells with dark cytoplasm and poorly visible nuclei | 13/22 (59%) |
|  | Highly reflective epithelial cell nuclei | 7/22 (32%) |
|  | Heterogeneity in size of epithelial cells | 6/22 (27%) |
|  | “White” cells present in-between epithelial cell borders | 4/22 (18%) |
|  | “Bullae” within epithelium | 6/22 (27%) |
|  | “Conjunctival-appearance” of corneal epithelium | 2/22  (9%) |
| **Bowman’s layer**  **(observed in 22 patients’ imagesets)** | Dendritiform cells with large cell bodies with longer processes (separate from adjacent cells) | 10/22 (45%) |
|  | Small dendritiform cells present with slightly elongated cellular processes, often touching adjacent cells | 8/22 (36%) |
|  | “spider web”-like Bowman’s layer appearance | 3/22 (14%) |
| **Corneal Nerves**  **(Basal plexus region observed in 22 patients’ imagesets)** | No visible basal plexus nerves / basal nerves absent | 9/22 (41%) |
|  | Beading of basal plexus nerves | 6/22 (27%) |
| **Stroma (observed in 23 patients’ imagesets)** | Activated keratocyte | 18/23 (78%) |
|  | Curvilinear connections between adjacent keratocyte nuclei | 13/23 (56%) |
|  | “Microtubules” connecting adjacent activated keratocytes | 12/23 (52%) |
|  | “Spindle” linear structures | 7/23  (30%) |
|  | Prominent stromal corneal nerves visible | 5/23 (22%) |
|  | “Ghost” keratocyte appearance (absence of nuclei, wide cellular processes, interconnected processes of adjacent cells in honeycomb appearance) | 4/23 (17%) |
|  | Round objects visible along keratocyte processes (honeycomb appearance) | 1/23  (4%) |
| **Endothelium (observed in 3 patients’ imagesets)** | White cells in endothelium | 2/3  (67%) |
|  | Abnormal endothelium with hyper-reflective nuclei | 1/3  (33%) |
|  | Normal endothelial cell appearance | 1/3  (33%) |
